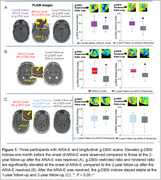# Early Detection and Monitoring of Amyloid‐Related Imaging Abnormalities in Monoclonal Antibody Therapy Using Generalized Diffusion Basis Spectrum Imaging (g‐DBSI): A Pilot Study

**DOI:** 10.1002/alz70862_109981

**Published:** 2025-12-23

**Authors:** Meng Jiang, Nelly Joseph‐Mathurin, Yong Wang, Brian A. Gordon, Carlos Cruchaga, Jason J. Hassenstab, Laura Ibanez, Yan Li, Richard J. Perrin, Alan E. Renton, Guoqiao Wang, Chengjie Xiong, Jorge J. Llibre‐Guerra, David B. Clifford, Alireza Atri, Eric McDade, Randall J. Bateman, Tammie L.S. Benzinger, Qing Wang

**Affiliations:** ^1^ Washington University School of Medicine, St. Louis, MO USA; ^2^ Washington University in St. Louis School of Medicine, St. Louis, MO USA; ^3^ Knight Alzheimer's Disease Research Center, Washington University in St. Louis, St. Louis, MO USA; ^4^ Mallinckrodt Institute of Radiology, Washington University School of Medicine, St. Louis, MO USA; ^5^ The Charles F. and Joanne Knight Alzheimer Disease Research Center, St Louis, MO USA; ^6^ Department of Neurology, Washington University School of Medicine in St. Louis, St. Louis, MO USA; ^7^ Department of Neurology, Washington University School of Medicine, St. Louis, MO USA; ^8^ Washington University St. Louis, St. Louis, MO USA; ^9^ The Charles F. and Joanne Knight Alzheimer Disease Research Center, Washington University, St. Louis, MO USA; ^10^ Department of Neurology, Washington University in St. Louis School of Medicine, St. Louis, MO USA; ^11^ Department of Genetics and Genomic Sciences, Icahn School of Medicine at Mount Sinai, New York, NY USA; ^12^ Ronald M Loeb Center for Alzheimer’s Disease, Department of Genetics & Genomic Sciences, Icahn School of Medicine at Mount Sinai, New York, NY USA; ^13^ Knight Alzheimer's Disease Research Center, St. Louis, MO USA; ^14^ Washington University School of Medicine in St. Louis, St. Louis, MO USA; ^15^ Banner Health, Phoenix, AZ USA; ^16^ Knight Alzheimer Disease Research Center, Washington University School of Medicine, St. Louis, MO USA; ^17^ Hope Center for Neurological Disorders, Washington University School of Medicine, St. Louis, MO USA; ^18^ Knight Alzheimer Disease Research Center, Saint Louis, MO USA

## Abstract

**Background:**

Amyloid‐Related Imaging Abnormalities (ARIA), characterized by vasogenic edema or sulcal effusions (ARIA‐E) or hemorrhagic lesions (microhemorrhages, superficial siderosis, or, rarely, microhemorrhages) (ARIA‐H), remain a significant challenge in monoclonal antibody (mAb) therapy for AD (1). Despite their clinical importance, the mechanisms underlying ARIA, particularly neuroinflammation, remain poorly understood. Advanced imaging techniques are crucial for elucidating these processes. Generalized diffusion basis spectrum imaging (g‐DBSI), an advanced diffusion MRI method evolved from DBSI (2,3), can reveal microstructural changes linked to neuroinflammation and vasogenic edema in white and gray matter. This study aims to use g‐DBSI to investigate the microstructural alterations linked to neuroinflammation and vasogenic edema during the development and recovery of ARIA‐E in the context of mAb treatment.

**Method:**

This pilot study included three participants from the DIAN‐TU‐001 trial who received Gantenerumab (4) and underwent longitudinal g‐DBSI scans. ARIA assessment was conducted by a panel of trained neuroradiologists using an FDA grading scale and standardized structured reporting templates, as recommended by the American Society for Neuroradiology (5). Regions of interest (ROIs) were delineated on the FLAIR sequence in areas exhibiting ARIA‐E. Within these ROIs, we quantified the g‐DBSI‐derived restricted ratio (RR, a putative marker of inflammation) and hindered ratio (HR, a putative marker of vasogenic edema).

**Result:**

For the first participant, an elevated g‐DBSI‐derived RR and HR were observed one month before the onset of ARIA‐E in the third participant compared to the 2‐year follow‐up g‐DBSI (Figure 1A). For the second participant, the initial g‐DBSI scan during ARIA‐E onset showed elevated RR and HR, which decreased significantly in the follow‐up scan two years later (Figure 1B). For the third participant, g‐DBSI‐derived RR and HR remained stable between 1‐ and 2‐year follow‐ups after the resolution of ARIA‐E (Figure 1C).

**Conclusion:**

Our findings suggest neuroinflammation’s role in ARIA‐E progression and demonstrate the potential of g‐DBSI to detect microstructural changes before the onset of ARIA‐E. This pilot study supports g‐DBSI as a noninvasive imaging tool to monitor ARIA progression, providing insights into neuroinflammation and vasogenic edema. Further research with larger cohorts is necessary to further delineate and validate the potential clinical utility of g‐DBSI.